# Mitral annular disjunction distance is associated with adverse outcomes in children and young adults with connective tissue disorders

**DOI:** 10.1016/j.jocmr.2025.101954

**Published:** 2025-09-06

**Authors:** Daniel A. Castellanos, Spencer B. Barfuss, Noah DiBiasio-Hudson, Grace Lee, Elizabeth DeWitt, Edward T. O’Leary, Lynn A. Sleeper, Chrystalle Katte Carreon, Stephen P. Sanders, Daniel Quiat, Michael N. Singh, Sunil J. Ghelani, Ronald V. Lacro

**Affiliations:** a Department of Cardiology, Boston Children's Hospital, Boston, Massachusetts, USA; b Department of Pediatrics, Harvard Medical School, Boston, Massachusetts, USA; c The Stella and Richard Van Praagh Cardiac Registry, Departments of Cardiology, Pathology, and Cardiac Surgery, Boston Children’s Hospital, Boston, Massachusetts, USA; d Department of Pathology, Harvard Medical School, Boston, Massachusetts, USA; e Department of Medicine, Harvard Medical School, Boston, Massachusetts, USA

**Keywords:** Congenital heart disease, Mitral annular disjunction, Connective tissue disorders, Marfan syndrome, Loeys-Dietz syndrome, Ehlers-Danlos syndrome

## Abstract

**Introduction:**

Mitral annular disjunction (MAD) is a pathologic fibrous separation of the mitral valve hinge point from the ventricular myocardium. The aims of this study were to describe the range of MAD distance by cardiovascular magnetic resonance (CMR) in children and young adults with connective tissue disorders (CTDs) versus a healthy control sample, and to assess the MAD distance as a predictor of adverse cardiovascular outcomes.

**Methods:**

This was a retrospective, single-center study of healthy subjects and patients with Marfan syndrome, Loeys-Dietz syndrome, Ehlers-Danlos syndrome, or nonspecific CTD who underwent CMR between January 01, 2000 and January 01, 2020. The MAD distance was measured from the 2-chamber, 4-chamber, and left ventricular outflow tract views in systole and diastole and analyzed as absolute values as well as indexed to BSA and height. The primary outcome was a composite defined as the presence of significant ventricular arrhythmias, cardiac arrest, and/or death. Age-adjusted odds ratios with 95% confidence intervals and c-statistic are reported. Classification and Regression Tree analysis was performed to identify the most discriminating binary threshold to predict the occurrence of the composite outcome.

**Results:**

Around 30 healthy control subjects and 254 patients with CTD met inclusion criteria. The mean ± SD age at initial CMR was 17 ± 6 years for patients with CTD and 14 ± 3 years for controls. The mean MAD distance was larger in patients with CTD compared to the control sample, and the maximum MAD distance in the control sample was 3.6 mm. Median follow-up in the CTD group was 5 years (IQR 3–11 years). Thirty-four (15%) patients met the composite outcome. Systolic MAD distance was positively associated with the composite outcome. The optimal binary threshold for height-indexed maximum systolic MAD distance was 0.033 mm/cm with an event rate of 18.6% at/above threshold versus 2.6% below threshold (AUC 0.74). The association was independent of other important clinical predictors.

**Conclusion:**

A small MAD distance can be measured in healthy children and young adults. Children and young adults with CTD have a longer MAD distance than healthy control subjects, and a longer MAD distance is associated with adverse outcomes.

## Graphical abstract

**Figure:**
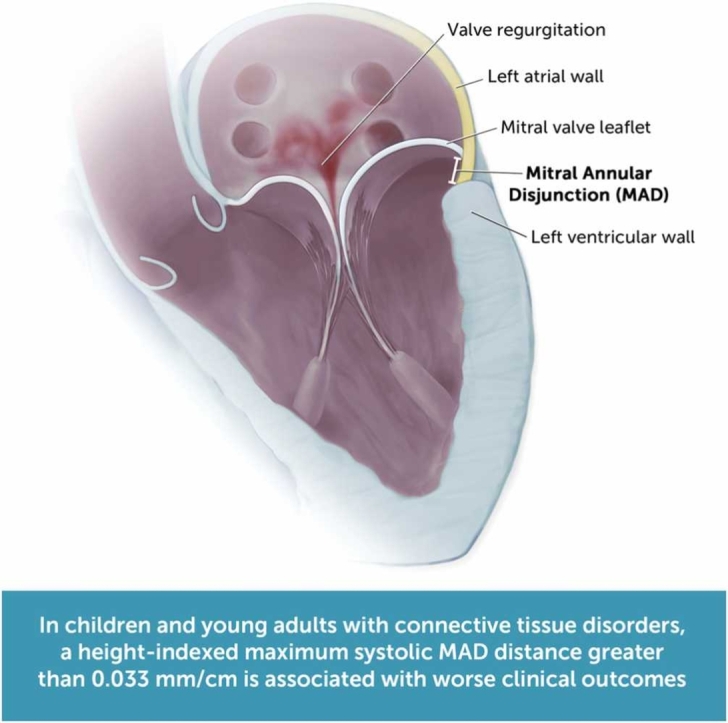
MAD in children and young adults. In children and young adults with CTDs, a height-indexed value of 0.033 mm/cm for the maximum systolic MAD distance was the best binary discriminator for patients with a composite outcome of significant ventricular arrhythmia, cardiac arrest, or death. *MAD* Mitral annular disjunction, *CTD* connective tissue disorders

## 1. Introduction

Mitral annular disjunction (MAD) is a pathologic fibrous separation of the mitral valve hinge point from the ventricular myocardium, which can be identified on cardiovascular magnetic resonance (CMR) by the longitudinal distance between the basal aspect of the left ventricular myocardium and the mitral valve hinge point (**Video 1**). This distance, often referred to as the MAD distance, has been described as an absolute value in publications of adults with MAD [1]. Studies largely based in adult populations, have found that patients with MAD have a significantly higher burden of arrhythmia [2]. Studies about MAD with significant numbers of pediatric patients have primarily been limited to Marfan syndrome [3], [4], and a larger MAD distance was associated with an increased rate of prophylactic aortic surgery and a higher incidence of ventricular ectopy [4]. MAD has not been examined in healthy children or in children with connective tissue disorders (CTD) other than Marfan syndrome. The ability of CMR to assess MAD in children and young adults has not been clearly shown. The purpose of this study was first to describe the range of MAD distance by CMR in children and young adults with CTD and in healthy control subjects and then assess ways in which the MAD distance can be represented in children and young adults such that it discriminates between patients with and without adverse clinical outcomes.

## 2. Methods

**Fig. 1:**
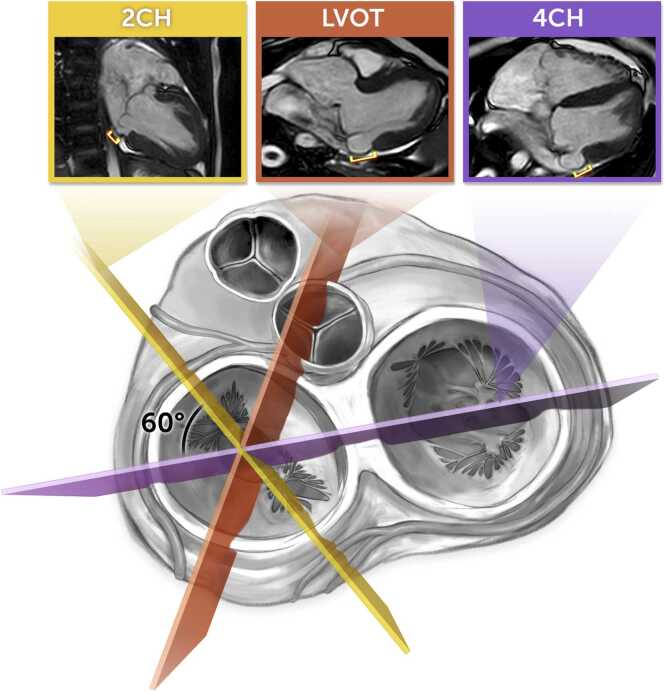
Example of MAD measurements. Systolic images from the 2-chamber, LVOT, and 4-chamber views of a CMR examination are shown at the top of the figure. The MAD distance is demonstrated with brackets. The bottom of the figure illustrates where each imaging plane overlies the heart. The MAD distances obtained were 8 mm, 15 mm, and 10 mm in the 2-chamber, LVOT, and 4-chamber views, respectively. *MAD* mitral annular disjunction, *LVOT* left ventricular outflow tract, *CMR* cardiovascular magnetic resonance

This was a retrospective, single-center study of patients with CTD and healthy subjects less than 30 years of age who underwent CMR with cine imaging at Boston Children’s Hospital between January 1, 2000, and January 1, 2020. MAD measurements were made on commercially available CMR analysis software (Circle Cardiovascular Imaging, cvi42, Version 5.14.2, Calgary, Alberta, Canada) during both systole and diastole on the 2-chamber (2CH), 4-chamber (4CH), and left ventricular outflow tract (LVOT) views (three routine cine imaging views, Fig. 1). Furthermore, given fewer data on how to analyze the MAD distance over the range of body sizes in children and young adults, the MAD distance was analyzed as an absolute value, indexed to BSA, and indexed to height (a total of 24 predictor variable candidates). Interobserver reliability for the MAD distance was assessed by a second blinded observer in 90 randomly selected CMR examinations in patients with CTD. The primary observer (measuring MAD in all patients) had 4 years of cardiovascular MRI experience and the second observer for the subset of 90 patients had 1 year of cardiovascular MRI experience. Other data reviewed included clinical notes, imaging reports, results of genetic evaluations and genetic testing, Holter monitor results, operative notes, and pathology reports.

Due to this study’s duration, the technical details for CMR acquisition changed over its course. At the beginning of the study (and for a small proportion of patients: 19%, 49/254), images were acquired on a 1.5T GE Signa scanner (GE Healthcare, Waukesha, Wisconsin). On this scanner fast gradient echo cine images were acquired, typically with the following parameters: TR of 13.6 ms, TE of 7.7 ms, flip angle of 15°, acquisition resolution of 1.09 mm × 2.18 mm, 5 mm slice thickness, and temporal resolution of 9–15 phases/cycle. However, most images were acquired on a 1.5T Philips Intera Achieva scanner (Philips Healthcare, Best, the Netherlands). Balanced steady-state free precession cine images were acquired on this scanner with the following typical parameters: TR 2.4 ms, TE 1.2 ms, flip angle 60°, acquired voxel size 1.8 × 1.8 × 8 mm^3^, compressed sensing-sensitivity encoding acceleration factor of 3, and temporal resolution 20 phases/cycle reconstructed to 30 phases/cycle. Imaging throughout the study period was most often performed with breath-holding at end-expiration.

The healthy control subjects were individuals referred for CMR due to a family history of cardiomyopathy (including hypertrophic cardiomyopathy and arrhythmogenic right ventricular dysplasia), sudden cardiac death, and arrhythmia. The CMR results for these patients were required to demonstrate normal ventricular volumes and function (including on follow-up CMR examinations, if performed) and no late gadolinium enhancement (LGE, including on follow-up CMR examinations, if performed). In addition, the control subjects must not have had congenital heart disease, prior surgeries, or arrhythmia on Holter monitor (if performed). The CTD cohort included patients with Marfan syndrome, early onset, severe (“neonatal”) Marfan syndrome, Loeys-Dietz syndrome, vascular Ehlers-Danlos syndrome (vEDS), Ehlers-Danlos syndrome (excluding vEDS), or nonspecific CTD. Patients in the non-vEDS Ehlers-Danlos group included patients with a nonspecific diagnosis of Ehlers-Danlos syndrome without specific genetic mutation or clinical diagnosis.

The primary outcome was a composite measure defined as the presence of significant ventricular arrhythmias, cardiac arrest, and/or all-cause mortality. Significant ventricular arrhythmias included cases with symptomatic ventricular tachycardia, sustained ventricular tachycardia, and automated implantable cardioverter defibrillator placement. It excluded cases of isolated post-operative ventricular tachycardia that did not require long-term management and asymptomatic non-sustained ventricular tachycardia on rhythm monitors. This study was approved by the Institutional Review Board of Boston Children’s Hospital.

### 2.1. Statistics

For all patients, MAD distances were analyzed as absolute, body surface area (BSA)-indexed, and height (ht)-indexed values. Descriptive statistics are presented as mean ± standard deviation (SD) for continuous measures with approximately a normal distribution and median (interquartile range) for other continuous or ordinal measures. Categorical data are described as frequency and percentage. When comparing MAD measurements among different CMR views, absolute and square-root transformed (variance-stabilizing/normalized) measurements were analyzed. The Wilcoxon rank sum test and analysis of covariance with height as the covariate were used to compare MAD measurements between patients with CTD and healthy control subjects. A key predictor variable for the remainder of the analysis, available for a subset of subjects, was the maximum MAD distance among all three of the standard CMR views (2CH, 4CH, and LVOT). Age-adjusted odds ratios with 95% confidence interval and c-statistic are reported based on logistic regression of the binary composite outcome on MAD distance with age at first CMR as the covariate. Odds ratios for the composite outcome as a function of height-indexed MAD are reported per 0.005 mm/cm increase. Classification and Regression Tree analysis was performed to identify the most discriminating binary MAD distance threshold to predict the occurrence of the composite outcome. The selection criterion for the binary split was based on maximizing the reduction in entropy. Stepwise logistic regression with MAD fixed in the model was used to assess whether MAD is an independent predictor of the composite outcome; covariate-adjusted odds ratios are reported. A p-value <0.05 was considered to be statistically significant. Analyses were performed with SAS 9.4 (SAS Institute, Inc., Cary, North Carolina) and R 4.1.2 [5]. The SAS code for selected analyses is included in the supplementary data.

For interobserver reliability, two-way mixed effects models were fit to estimate the intraclass correlation coefficient (ICC). Furthermore, to assess for the effect of era, interobserver reliability was assessed in an earlier versus later era (on or before December 31, 2005 versus January 01, 2006 or later, which approximately reflects the transition from the 1.5T GE Signa scanner to the 1.5T Philips Intera Achieva scanner). Values <0.5 indicate poor reliability, values between 0.5–0.75 indicate moderate reliability, values between 0.75–0.9 indicate good reliability, and values >0.90 indicate excellent reliability [6].

## 3. Results

Thirty healthy control subjects and 254 patients with CTD met inclusion criteria. Over half of the patients with CTD had Marfan syndrome (53.1%, 135/254). The mean age at initial CMR was 17 ± 6 years for patients with CTD and 14 ± 3 years for control subjects (p = 0.006). Mean height was also greater for the patients with CTD relative to the control group (Table 1). Despite the differences in age and height, there was no difference in weight or BSA (Table 1). One hundred twenty-one patients (48%) with CTD were diagnosed with mitral valve prolapse (MVP), while none of the patients in the control group had MVP.

**Table 1:** Demographic characteristics for patients with CTDs and healthy control subjects.

	Healthy			Connective Tissue Disorders			
	N	Mean ± SD	Median (range)	N	Mean ± SD	Median (range)	p-value
Age, y	30	14.3 ± 3.9	13.5 (5, 22)	254	17.1 ± 6.9	18.4 (0.2, 29.7)	0.006
Wt, kg	30	56.0 ± 15.3	58.3 (28.3, 99.9)	254	60.3 ± 26.6	61.7 (2.3, 155.6)	0.20
Ht, cm	30	150.2 ± 38.6	162.0 (15.1, 179.0)	253	165.8 ± 29.9	174.5 (46.0, 210.8)	<0.001
BSA, m^2^	30	1.57 ± 0.27	1.64 (1.03, 2.17)	254	1.64 ± 0.51	1.73 (0.17, 2.93)	0.08

*CTD* connective tissue disorders, *SD* standard deviation, *BSA* body surface area, *ht* height, *wt* weight

In both the control and CTD groups, there was no difference in mean systolic MAD according to view (2CH vs. 4CH vs. LVOT, Supplemental Tables 1 and 2). For diastolic MAD measurements in the control group, the 2CH view was longer than the LVOT view (p = 0.029), and the 4CH and LVOT views were similar to each other (p = 0.69). For diastolic MAD measurements in the group with CTD, the 2CH view was longer than both than the 4CH (p<0.001) and LVOT (p = 0.001) views, and the 4CH and LVOT views were similar to each other (p = 0.95). The maximum MAD distance in the control group was 3.6 mm. For the patients with CTD, the MAD distance was larger in all standard CMR views compared to the control group (Table 2). For many measures, there was a weak positive correlation between MAD measurement and both age and height, but no correlation with BSA (Supplemental Table 3). There was a moderate positive correlation between MAD measurement and mitral valve size.

**Table 2:** Mean MAD distance (mm) in patients with CTD and healthy control subjects.

	Mean ± SD			Height-adjusted Mean ± SE		
Variable	Control	CTD	p-value	Control	CTD	p-value
2CH systole	0.6 ± 1.0	3.5 ± 4.0	<0.001	0.84 ± 0.71	3.51 ± 0.3	<0.001
2CH diastole	0.5 ± 1.1	1.5 ± 2.2	0.02	0.58 ± 0.40	1.47 ± 0.15	0.04
4CH systole	0.3 ± 0.7	3.2 ± 3.6	<0.001	0.46 ± 0.63	3.18 ± 0.24	<0.001
4CH diastole	0.2 ± 0.6	0.8 ± 1.7	0.03	0.24 ± 0.30	0.80 ± 0.11	0.08
LVOT systole	0.2 ± 0.7	3.4 ± 4.2	<0.001	0.43 ± 0.94	3.43 ± 0.27	0.002
LVOT diastole	0.1 ± 0.2	0.9 ± 1.8	0.01	0.12 ± 0.40	0.93 ± 0.11	0.05

*MAD* mitral annular disjunction, *CTD* connective tissue disorders, *2CH* two-chamber view, *4CH* four-chamber view, *LVOT* left ventricular outflow tract view

LGE imaging was performed in 49 of the patients with CTD. Only three cases had left ventricular LGE. In one of these cases, LGE was noted at the anterolateral papillary muscle; in the other two cases, LGE was noted along the interventricular septum. Given the small number of cases with LGE, this variable was not included in the clinical outcome analysis.

### 3.1. Relationship of the MAD distance with the composite outcome

The median follow-up was 5 years (interquartile range [IQR] 3–11 years) from the first CMR examination. Twenty‐one (8.3%) of the patients with CTD met the composite outcome with a mean age of 13.3 ± 8.1 years at CMR (versus 17.4 ± 18.6 years at CMR in those without the composite outcome, p = 0.008). Exactly 2 patients (0.8%) had a significant ventricular arrhythmia, 5 patients (2.0%) had a cardiac arrest, and 18 patients (7.1%) died. There were no cases of heart transplant, and 3 patients could not be resuscitated after cardiac arrest (thus meeting both the cardiac arrest and death endpoints).

The MAD distance was generally larger in patients for whom the composite outcome occurred, whether assessed as an absolute, BSA-indexed, or height-indexed value (Table 3). The maximum systolic MAD distance, in patients where an MAD distance was measured in all 3 CMR views, was the most discriminating measure, even though the goodness-of-fit was still modest (c-statistic of 0.70–0.74). The MAD distances that were most predictive of the composite outcome (using a binary threshold) were the maximum systolic MAD measurement (area under the receiver-operator curve [AUC] of 0.70), the BSA-indexed maximum systolic MAD measurement (AUC 0.70), and the height-indexed maximum systolic MAD measurement (AUC 0.74) among cases in which all three CMR views were measurable (Table 4). Operating characteristics for the absolute, BSA-indexed, and height-indexed maximum systolic MAD distance among all views are shown in Supplemental Table 4. For a height-indexed maximum systolic MAD threshold of 0.033 mm/cm, the sensitivity was 79% (11/14), specificity was 70% (111/159), accuracy was 71% (122/173), positive predictive value was 19% (11/59), and negative predictive value was 97% (111/114, **Graphical Abstract**). While the cases with an outcome event were more likely to have a longer MAD distance than those without an outcome event, there were cases with an event that did not have MAD (Supplemental Figure 1).

**Table 3:** Logistic regression of the binary composite outcome with MAD distance measurements.

MAD measurement	Mean MAD measurement				Age-adjusted Odds Ratio (95% CI)	p-value	c-statistic
	No event (N = 233)		Event (N = 21)				
	N	Mean	N	Mean			
*Absolute value (mm)*							
2CH systolic	175	3.35	19	5.42	1.14 (1.03, 1.26)	**0.013**	0.72
4CH systolic	181	3.14	16	3.89	1.09 (0.95, 1.24)	0.22	0.68
LVOT systolic	217	3.27	18	5.47	1.14 (1.03, 1.26)	**0.013**	0.72
Max systolic among 3 views	159	4.80	14	7.02	1.14 (1.01, 1.27)	**0.028**	0.74
2CH diastolic	175	1.43	19	1.98	1.16 (0.95, 1.41)	0.15	0.69
4CH diastolic	181	0.85	16	0.41	0.86 (0.55, 1.34)	0.50	0.67
LVOT diastolic	217	0.87	18	1.75	1.26 (1.03, 1.53)	**0.026**	0.72
Max diastolic among 3 views	160	2.06	14	2.55	1.17 (0.93, 1.46)	0.17	0.72
*BSA-indexed (mm/m^2^)*							
2CH systolic	175	2.16	19	4.79	1.17 (1.02, 1.33)	**0.022**	0.72
4CH systolic	180	2.02	16	4.75	1.15 (1.01, 1.30)	**0.031**	0.68
LVOT systolic	217	2.03	18	6.22	1.17 (1.05, 1.30)	**0.005**	0.71
Max systolic among 3 views	159	3.11	14	7.75	1.16 (1.03, 1.30)	**0.014**	0.75
2CH diastolic	175	0.90	19	1.24	1.15 (0.86, 1.53)	0.36	0.67
4CH diastolic	181	0.52	16	0.30	0.83 (0.45, 1.54)	0.56	0.66
LVOT diastolic	217	0.53	18	1.67	1.39 (1.07, 1.79)	**0.013**	0.72
Max diastolic among 3 views	160	1.29	14	2.04	1.23 (0.93, 1.64)	0.15	0.71
*Ht-indexed (mm/cm)*							
2CH systolic	175	0.020	19	0.031	1.11 (1.03, 1.21)	**0.009**	0.73
4CH systolic	180	0.019	16	0.038	1.08 (0.99, 1.18)	0.076	0.67
LVOT systolic	216	0.019	18	0.043	1.10 (1.03, 1.18)	**0.007**	0.72
Max systolic among 3 views	159	0.029	14	0.054	1.10 (1.02, 1.19)	**0.018**	0.75
2CH diastolic	175	0.009	19	0.012	1.11 (0.95, 1.31)	0.19	0.69
4CH diastolic	181	0.005	16	0.003	0.88 (0.61, 1.27)	0.50	0.67
LVOT diastolic	216	0.005	18	0.013	1.22 (1.04, 1.43)	**0.014**	0.72
Max diastolic among 3 views	160	0.012	14	0.017	1.15 (0.96, 1.37)	0.13	0.72

Note that odds ratios for height-indexed MAD distances are per increase in 0.005 mm/cm. *BSA* body surface area, *CI* confidence interval, *Ht* height, *MAD* mitral annular disjunction, *2CH* two-chamber view, *4CH* four-chamber view, *LVOT* left ventricular outflow tract view

**Table 4:** Optimal binary threshold for MAD measurements to predict the composite outcome.

Predictor	Cutoff value	N	Event rate below (%)	N	Event rate at or above (%)	AUC
*Absolute value (mm)*						
2CH diastole	1.867	128	7.0%	66	15.2%	0.60
4CH diastole	2.725	170	9.4%	27	0%	0.57
LVOT diastole	2.925	210	5.7%	25	24.0%	0.62
Max diastole among 3 views	1.867	85	4.7%	89	11.2%	0.61
2CH systole	2.878	101	4.0%	93	16.1%	0.67
4CH systole	1.299	74	4.1%	122	10.7%	0.60
LVOT systole	2.988	140	2.9%	95	14.7%	0.70
Max systole among 3 views	5.587	113	3.5%	60	16.7%	0.70
*BSA-indexed (mm/m^2^)*						
2CH diastole	2.304	160	7.5%	34	20.6%	0.60
4CH diastole	1.119	159	9.4%	38	2.6%	0.57
LVOT diastole	1.745	202	5.0%	33	24.2%	0.66
Max diastole among 3 views	2.304	131	5.3%	43	16.3%	0.64
2CH systole	2.136	112	3.6%	82	18.3%	0.70
4CH systole	14.490	194	7.2%	2	100%	0.56
LVOT systole	9.084	226	5.8%	9	55.6%	0.63
Max systole among 3 views	2.555	89	2.3%	84	14.3%	0.70
*Height-indexed (mm/cm)*						
2CH diastole	0.022	161	8.1%	33	18.2%	0.58
4CH diastole	0.020	179	8.9%	18	0%	0.55
LVOT diastole	0.019	211	5.2%	23	30.4%	0.66
Max diastole among 3 views	0.032	160	6.9%	14	21.4%	0.57
2CH systole	0.016	94	2.1%	100	17.0%	0.71
4CH systole	0.095	193	7.3%	3	66.7%	0.56
LVOT systole	0.027	165	3.6%	69	17.4%	0.70
Max systole among 3 views	0.033	114	2.6%	59	18.6%	0.74

*2CH* two-chamber view, *4CH* four-chamber view, *LVOT* left ventricular outflow tract view

The height-indexed maximum systolic MAD distance was longer in CTD patients with MVP (0.045 ± 0.034 in those with MVP versus 0.016 ± 0.012 mm/cm in those without MVP, p<0.001). Additionally, height-indexed maximum systolic MAD was a predictor of the composite outcome independent of MVP, which was not significant in a bivariate model with MAD. The odds ratio for height-indexed maximum systolic MAD was 12.50 (95% CI 2.62, 59.54) while the odds ratio for MVP was 2.02 (95% CI 0.49, 8.25).

Given the above results, height-indexed maximum systolic MAD with a threshold of 0.033 mm/cm was evaluated in a covariate-adjusted logistic regression model that included other predictors of the composite outcome (c-stat of 0.91, Table 5). This model demonstrated that height-indexed maximum systolic MAD was an independent predictor of the composite clinical outcome, with an odds ratio of 11.79 (95% CI 2.24, 62.01) and p-value of 0.004. Other independent predictors included in the model were age at first CMR, history of aortic valve surgery, burden of ventricular ectopy, and diagnosis of non-vEDS Ehlers-Danlos syndrome. The addition of maximum aortic root and ascending aorta Z-scores to the model (not significant, p>0.45) did not alter the significance of height-indexed maximum systolic MAD as a predictor of the composite outcome. Univariate model results for all candidate clinical predictors are shown in Supplemental Table 5.

**Table 5:** Covariate-adjusted logistic regression for the composite outcome (N = 163).

Variable	Odds ratio (95% CI)	p-value
*Univariate model, c-stat = 0.74*		
Ht-indexed max systolic MAD among 3 views*	8.48 (2.26, 31.76)	0.002
*Covariate-adjusted model, c-stat = 0.91*		
Ht-indexed max systolic MAD among 3 views*	11.79 (2.524, 62.01)	0.004
Age at CMR, y	0.90 (0.81, 0.99)	0.024
Aortic valve surgery	6.77 (1.57, 29.31)	0.011
Max VPB	1.23 (1.06, 1.43)	0.006
Ehlers-Danlos syndromes (excluding vEDS)	10.20 (1.54, 67.53)	0.016
*as a binary predictor with ≥0.033 mm/cm		

Patients in which MAD could not be measured in all 3 views were excluded. Note that when the maximum aortic root and ascending aorta Z-scores were added to the model both were not significant and did not alter the significance of height-indexed maximum systolic MAD as a predictor of the composite outcome. *CMR* cardiovascular magnetic resonance, *Ht* height, *MAD* mitral annular disjunction, *vEDS* vascular Ehlers-Danlos syndrome, *VPB* ventricular premature beats

### 3.2. Comparing height-indexed MAD distances and the composite outcome by genetic diagnosis

There was a significant difference in height-indexed MAD distance among the 6 CTD diagnostic groups (p = 0.003). The mean height-indexed MAD distances were similar between Marfan and Loeys-Dietz syndromes and longer than the other genetic groups (Table 6). Although there were only 7 patients with early onset, severe Marfan syndrome, their height-indexed MAD distances were longer than other groups (0.091 ± 0.065 versus 0.029 ± 0.025 mm/cm, p<0.001) and longer than the patients with classical Marfan syndrome (median 0.092 IQR 0.042, 0.137 versus 0.026 IQR 0.014, 0.047 mm/cm, p = 0.023). The composite event rates were similar among the 6 CTD diagnostic groups (p = 0.26) but higher when comparing early onset, severe Marfan syndrome to all other CTD patients (57.1%, 4/7, versus 6.9%, 17/247; p = 0.001). The one patient within the group of CTD classified as “other” who died had multi-system smooth muscle dysfunction syndrome (with a mutation in *ACTA2*). The 3 patients in the non-vEDS Ehlers-Danlos group who met the composite outcome all had atypical CTD presentations and carried a nonspecific diagnosis of Ehlers-Danlos syndrome without specific genetic mutation or clinical diagnosis (i.e., specific type of EDS).

**Table 6:** Average MAD distance and frequency of composite outcome by CTD diagnosis.

CTD Diagnosis	MAD distance (mm/cm)	p-value for MAD distance	Composite event rate (%)
Marfan syndrome (N = 135)	0.036 ± 0.033	<0.001	11 (8.2%)*
Loeys-Dietz syndrome (N = 41)	0.030 ± 0.025	<0.001	5 (12.2%)
Ehlers-Danlos syndromes (excluding vEDS) (N = 20)	0.019 ± 0.023	0.006	3 (15.0%)
vEDS (N = 7)	0.003 ± 0.006	0.42	0 (0%)
Nonspecific CTD (N = 45)	0.022 ± 0.021	<0.001	1 (2.2%)
Other (N = 6)	0 ± 0		1 (16.7%)
*The composite event rate was 57.1% (4/7) for early onset, severe Marfan syndrome compared to 5.5% (7/128) for classical Marfan syndrome.			

Values are mean ± standard deviation or count (%). The composite event rates were similar among the 6 CTD diagnostic groups (p = 0.26). The six patients in the other category (with one patient per diagnosis) include: Filamin A (*FLNA*) mutation, *ACTA2* R179 multi-system smooth muscle dysfunction syndrome, arterial tortuosity syndrome, *ADAMTSL4*, bicuspid aortic valve, and chromosome 2 deletion involving *COL3A1*, *COL5A2*, and *TTN*. The patient with *ACTA2* was the only one to meet the composite outcome in this group. *CTD* connective tissue disorder, *MAD* mitral annular disjunction, *vEDS* vascular Ehlers-Danlos syndrome

### 3.3. Change in MAD distance over serial CMR examinations

There was a mean duration of 7 years between initial and most recent CMR examinations. Systolic MAD distances could only be measured in all three views on the initial and most recent CMR examinations in 34 patients. In those examinations, the height-indexed MAD did not change between the two time points, with a median change of systolic MAD distance of 0 mm/cm (IQR −0.005 to 0.007 mm/cm, p-value 0.65).

### 3.4. Interobserver reliability

There is very good interobserver agreement of the systolic MAD distance in the LVOT view (ICC: 0.89, 95% CI: 0.86–0.91; Supplemental Table 6). There is good interobserver agreement of the systolic MAD distance in the 4CH view (ICC: 0.81, 95% CI: 0.76–0.85) and both the systolic (ICC: 0.76, 95% CI: 0.71–0.81) and diastolic (ICC: 0.73, 95% CI: 0.67–0.78) MAD distances in the 2CH view. There is moderate interobserver agreement of the diastolic MAD distance in the 4CH (ICC: 0.56, 95% CI: 0.47–0.64) and LVOT (ICC: 0.52, 95% CI: 0.43–0.61) views. Interobserver agreement was similar between the earlier and later eras in all views during systole and in the 2CH and 4CH views during diastole (Supplemental Table 7). Interobserver agreement was higher in the later era for the LVOT diastolic view.

### 3.5. MAD and CMR pathologic correlate

**Fig. 2:**
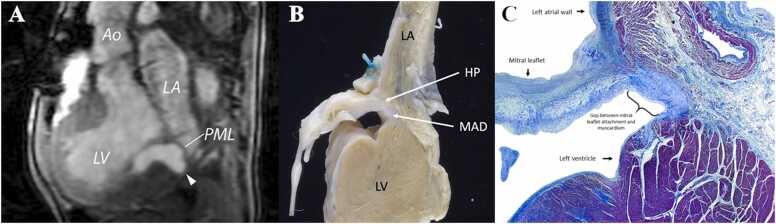
MRI and pathology correlate of MAD in a patient with early onset Marfan syndrome. A) Systolic still frame from fast gradient echo cine image in the LVOT view demonstrating MAD (6 mm separation of the posterior mitral leaflet hinge point from the basal-most aspect of the ventricular myocardium). Ao – aorta, LA – left atrium, LV – left ventricle, PML – posterior leaflet of the mitral valve; caret indicates region of mitral annular disjunction. B) Gross pathological specimen demonstrating evidence of MAD with fibrous separation of the mitral valve hinge point from the left ventricular myocardium. LA – left atrial wall, LV – left ventricular myocardium, HP – mitral valve hinge point, MAD – region of mitral annular disjunction. C) Masson trichrome stain at low power magnification (1.25×) showing the gap between the mitral leaflet attachment and the superior aspect of LV myocardium. The brace indicates the region of MAD. *MAD* mitral annular disjunction, *LVOT* left ventricular outflow tract, *LA* left atrial wall, *LV* left ventricular, *PML* posterior leaflet of the mitral valve, *HP* mitral valve hinge point

A heart specimen of a girl with early onset, severe Marfan syndrome who was included in this study was available for review. The patient had a valve-sparing aortic root replacement at age 6 months. The patient underwent a CMR examination at 3 years of age and died at 4 years of age. Findings at CMR included a dilated ascending aorta, severe aortic regurgitation, moderate-to-severe mitral regurgitation, a dilated left atrium and ventricle, and normal left ventricular systolic function. Six months after CMR, the patient underwent placement of a mechanical aortic valve, re-replacement of the aortic root, and mitral valvuloplasty. One month following surgery, the patient experienced cardiac arrest from which she could not be resuscitated. Gross and histopathologic review confirmed MAD (Fig. 2 clearly demonstrates the fibrous separation of the mitral valve hinge point from the left ventricular myocardium), as well as thrombosis of the aortic prosthesis.

## 4. Discussion

This study found that a small separation (up to 3.6 mm) between the mitral valve hinge point and ventricular myocardium can be observed in healthy children and young adults and is likely not indicative of adverse outcomes. By comparison, children and young adults with CTD have a longer MAD distance compared to healthy control subjects. This longer MAD distance is not limited to Marfan syndrome but also present in other CTDs, including various forms of Loeys-Dietz syndrome, atypical CTDs, and CTD without a specifically identified genetic mutation. In patients with CTD, longer absolute, BSA-indexed, and height-indexed systolic MAD distances were associated with the composite outcome of significant ventricular arrhythmia, cardiac arrest, or death. In these patients, a height-indexed value of 0.033 mm/cm for the maximum systolic MAD distance best discriminated between those with and without the composite outcome. Furthermore, in a covariate-adjusted model, the cutoff value of 0.033 mm/cm for the maximum systolic height-indexed MAD distance was an independent predictor of the composite outcome.

Patients with CTD are often referred to CMR to assess aortic dimensions, and it is a common practice at our center to obtain a limited set of cine images during these examinations. Since the maximum systolic MAD distance among the 2CH, 4CH, and LVOT views had a higher AUC relative to any of the individual cine views alone, we recommend performing each of the above cines in at least one CMR examination for patients with CTD. Notably, the 4CH view had the lowest odds ratios. Since the 4CH view visualizes the mitral valve close to the commissures, it is a suboptimal view to measure MAD distance. Additionally, the systolic measurements in the various views had superior interobserver reliability and more often had statistically significant associations with the composite outcome relative to diastolic MAD measurements. Therefore, for clinical studies, we advocate for measuring MAD distances during systole in all three CMR views (2CH, 4CH, and LVOT).

A recent study assessing the progression of MAD distance throughout childhood in patients with Marfan syndrome found that height-indexed MAD distance was constant over time [3]. In our study of patients with CTD, the height-indexed MAD distance was also constant over time and best discriminated between those with and without the composite outcome. However, the AUC, sensitivity, specificity, negative predictive value, positive predictive value, and accuracy for the absolute and BSA-indexed MAD distances were similar to those for the height-indexed MAD. In agreement with prior studies, we recommend using height-indexed MAD distances as the optimal method to describe MAD in children and young adults with CTD. More studies on the prognostic value of the MAD distance in children without CTD are needed.

Our study found that MAD is not limited to Marfan syndrome and was present in other forms of CTD. Furthermore, half of the composite outcome events in this study occurred in patients without Marfan syndrome, although the proportion of patients meeting the composite outcome was highest in those with early-onset Marfan syndrome. Prior studies have shown that patients with early onset Marfan syndrome have a high mortality in their first few years of life [7], which differs from the prognosis of classical Marfan syndrome [8], [9], [10]. Case series in patients with early onset Marfan syndrome have reported that valvular dysfunction (including mitral valve dysfunction) is common and may contribute to mortality in this patient population. MAD may play a role in the valvular pathology particularly for patients with early onset Marfan syndrome and more study on MAD in this population is required. Furthermore, this study demonstrates that CTD patients with MAD are at risk for life-threatening ventricular arrhythmias. Our data demonstrates that height-indexed MAD distance has a high negative predictive value in identifying patients at risk for the composite study outcome, possibly representing a useful factor to aid in ventricular tachycardia/sudden cardiac death risk stratification. Thus, this study’s findings can inform more accurate risk stratification and efficient use of diagnostic imaging in pediatric and young adult patients with CTD.

## 5. Limitations

The group of healthy control subjects was small and tended to be younger and shorter than the group with CTD, due to the strict inclusion criteria, which limited the number of control subjects in this retrospective study. However, a height-adjusted comparison of MAD in the CTD and control cohorts yielded nearly identical inferences (longer MAD in patients with CTD). The number of clinical events in the study was small, and resampling was not performed to assess for overfitting. Thus, the thresholds are exploratory and have not yet been validated in a larger cohort. A study cohort with more clinical events and the use of other classification approaches may improve the robustness of a threshold for MAD in predicting adverse outcomes. Our cohort is relatively young and will need careful longitudinal follow-up to assess the cumulative risk of adverse events over the long term. This study does not fully account for technical advances in CMR (such as improved spatial and temporal resolution) over the 20-year period for this study. Although the interobserver agreement for systolic views suggests adequate measurement reproducibility, there was improvement in interobserver agreement for MAD measurement in the LVOT diastolic view in the later era compared to the earlier era. Thus, it is unknown whether the maximum diastolic measurement could have had a stronger association with the composite outcome if the interobserver reliability data were more robust in the diastolic LVOT view. Other CMR sequences, such as those with feature tracking parameters, may be of value in this population but were not studied [11]. Also, the natural history of MAD in patients with CTD and the effect of medical therapy cannot be delineated given most of the cohort was on a cardiac medication. Finally, although the development of ventricular fibrosis identified by LGE has been attributed as the mechanism for arrhythmia in patients with MAD [12], [13], only a small subset of cases in our cohort had imaging for LGE, which limits insight into the role of ventricular fibrosis in childhood.

## 6. Conclusions

A small fibrous separation between the mitral valve hinge point and ventricular myocardium can be visualized by CMR in normal children and young adults. Children and young adults with CTD have a longer MAD distance than healthy control subjects. MAD and associated adverse cardiovascular outcomes were observed in patients with Marfan syndrome as well as in patients with Loeys-Dietz syndrome, atypical CTDs, and with CTDs without a specific genetic diagnosis. While the MAD distance can be assessed in the LVOT, 2CH, and 4CH views during both systole and diastole, the maximum distance among all three views during ventricular systole was the key predictor variable. A height-indexed value of 0.033 mm/cm for the maximum systolic MAD distance was the best binary discriminator for patients with a composite outcome of significant ventricular arrhythmia, cardiac arrest, or death from patients without these outcomes. Height-indexed MAD distance remained an independent predictor of the composite outcome in a covariate-adjusted model. The proportion of patients in this young cohort who met the composite outcome was relatively low. Careful longitudinal follow-up is needed to assess the cumulative risk of adverse events over the long term. Further study is needed to better understand the prognostic value of the MAD distance in children without CTD as well as on the management of children with MAD.

## Funding

The project was completed with no external funding support.

## Author contributions

**Daniel A. Castellanos:** Writing – review & editing, writing – original draft, project administration, methodology, formal analysis, data curation, conceptualization. **Spencer B. Barfuss:** Writing – review & editing, data curation. **Noah DiBiasio-Hudson:** Writing – review & editing, project administration, formal analysis, data curation. **Grace Lee:** Writing – review & editing, project administration, data curation. **Elizabeth DeWitt:** Writing – review & editing, methodology, data curation. **Edward T. O’Leary:** Writing – review & editing, methodology, data curation. **Lynn A. Sleeper:** Writing – review & editing, writing – original draft, methodology, formal analysis, conceptualization. **Chrystalle Katte Carreon:** Writing – review & editing, data curation. **Stephen P. Sanders:** Writing – review & editing, data curation. **Daniel Quiat:** Writing – review & editing, data curation. **Michael N. Singh:** Writing – review & editing, data curation. **Sunil J. Ghelani:** Writing – review & editing, data curation. **Ronald V. Lacro:** Writing – review & editing, writing – original draft, validation, supervision, methodology, data curation, conceptualization.

## Ethics approval and consent

The study was approved by the Institutional Review Board at Boston Children’s Hospital and the requirement for informed consent was waived.

## Consent for publication

Not applicable.

## Declaration of competing interests

The authors declare that they have no known competing financial interests or personal relationships that could have appeared to influence the work reported in this paper.

## Data Availability

The datasets used and/or analyzed during the current study are available from the corresponding author on reasonable request.

## Appendix A Supplementary material

.

Cine imaging representing mitral annular disjunction (MAD). A 6-year-old female with early onset Marfan (including *FBN1* deletion) underwent a CMR examination which included 2-chamber (A), left ventricular outflow tract (B), and 4-chamber (C) views. MAD is a fibrous separation of the mitral valve hinge point from the ventricular myocardium. In this patient, the abnormally increased distance between the posterior mitral valve hinge point and ventricular myocardium is most prominent during ventricular systole but can also be noted during ventricular diastole. The examination also noted mitral valve prolapse, severe mitral regurgitation (calculated regurgitant fraction of 52%), no late gadolinium enhancement (not pictured), a severely dilated aortic root, and a normal ascending aorta (not pictured). On a Holter performed the same year as this CMR the patient also had frequent isolated ventricular premature beats (generally monoform, calculated as 5% of all beats) and a 5-beat run of non-sustained ventricular tachycardia. Three months after CMR, she underwent mitral valvuloplasty, mitral annuloplasty, and tricuspid valvuloplasty with a De Vega annuloplasty followed by valve-sparing aortic root replacement three years later. The patient was alive at most recent follow-up, at age 11 years
