# Supplementary material for: Mitral annular disjunction distance is associated with adverse outcomes in children and young adults with connective tissue disorders
Source: J Cardiovasc Magn Reson. 2025 Sep 6;27(2):101954. doi: 10.1016/j.jocmr.2025.101954 (PMC12702100; doi:10.1016/j.jocmr.2025.101954)

**Supplemental** **Table 1**: Distribution of MAD measurements (in mm) by view in healthy control subjects

| View | Mean | Std Dev | Median | Min | 25th | 75th | Max | p-value |
| --- | --- | --- | --- | --- | --- | --- | --- | --- |
| 2CH systole | 0.6 | 1.0 | 0 | 0 | 0 | 1.4 | 2.7 | Ref |
| 4CH systole | 0.2 | 0.6 | 0 | 0 | 0 | 0 | 2.3 | 0.10 |
| LVOT systole | 0.2 | 0.7 | 0 | 0 | 0 | 0 | 2.6 | 0.10 |
| 2CH diastole | 0.7 | 1.2 | 0 | 0 | 0 | 1.5 | 3.6 | Ref |
| 4CH diastole | 0.2 | 0.7 | 0 | 0 | 0 | 0 | 3.2 | 0.05 |
| LVOT diastole | 0.1 | 0.2 | 0 | 0 | 0 | 0 | 1.1 | 0.029 |

For systole, p-value comparing all 3 views: p=0.19

For diastole, p-value comparing all 3 views: p=0.06

In the control sample, there is no difference in mean systolic MAD according to CMR view (for comparison of systolic LVOT and 4CH measurements: p-value of 0.95). For diastolic MAD measurements, the 2-chamber view is larger than the LVOT view. Otherwise, the diastolic LVOT and 4CH measurements are similar with a p-value of 0.69.

**Supplemental** **Table 2**: Distribution of MAD measurements (in mm) by view in patients with connective tissue disorders

| View | Mean | Std Dev | Median | Min | 25th | 75th | Max | p-value |
| --- | --- | --- | --- | --- | --- | --- | --- | --- |
| 2CH systole | 3.8 | 4.0 | 2.9 | 0 | 0 | 5.7 | 16.9 | Ref |
| 4CH systole | 3.3 | 3.5 | 2.5 | 0 | 0 | 4.9 | 16.2 | 0.22 |
| LVOT systole | 3.8 | 4.5 | 2.1 | 0 | 0 | 6.6 | 16.6 | 0.91 |
| 2CH diastole | 1.6 | 2.3 | 0 | 0 | 0 | 3.0 | 12.5 | Ref |
| 4CH diastole | 0.9 | 1.8 | 0 | 0 | 0 | 1.5 | 10.5 | <0.001 |
| LVOT diastole | 0.9 | 1.9 | 0 | 0 | 0 | 1.5 | 11.7 | 0.001 |

For systole, p-value comparing all 3 views: p=0.41

For diastole, p-value comparing all 3 views: p<0.001

In the CTD sample, there is no difference in mean systolic MAD according to CMR view (for comparison of systolic LVOT and 4CH measurements: p-value of 0.27). For diastolic MAD measurements, the 2-chamber view is significantly larger than the 4-chamber and LVOT views, which are similar (for comparison of diastolic LVOT and 4CH measurements: p-value of 0.95).

**Supplemental Table 3**: Spearman Correlation between MAD vs. age, body size measures, and mitral valve measurements in patients with connective tissue disorders (CTDs). No correlations were significantly different from zero unless noted with *.

| MAD measurements | Variable | R |
| --- | --- | --- |
| 2CH systole | Age | 0.14* |
| 2CH diastole | Age | 0.08 |
| 4CH systole | Age | 0.17* |
| 4CH diastole | Age | 0.16* |
| LVOT systole | Age | 0.15* |
| LVOT diastole | Age | 0.13 |
| 2CH systole | Weight | 0.12 |
| 2CH diastole | Weight | 0.14 |
| 4CH systole | Weight | 0.03 |
| 4CH diastole | Weight | 0.17* |
| LVOT systole | Weight | 0.06 |
| LVOT diastole | Weight | 0.07 |
| 2CH systole | Height | 0.19* |
| 2CH diastole | Height | 0.09 |
| 4CH systole | Height | 0.18* |
| 4CH diastole | Height | 0.15* |
| LVOT systole | Height | 0.18* |
| LVOT diastole | Height | 0.10 |
| 2CH systole | BSA | 0.13 |
| 2CH diastole | BSA | 0.12 |
| 4CH systole | BSA | 0.06 |
| 4CH diastole | BSA | 0.17 |
| LVOT systole | BSA | 0.08 |
| LVOT diastole | BSA | 0.07 |
| 2CH systole | MV systole | 0.55* |
| 2CH diastole | MV diastole | 0.23* |
| 4CH systole | MV systole | 0.53* |
| 4CH diastole | MV diastole | 0.34* |

*p<0.05

**Supplemental** **Table 4**: Operating characteristics for maximum of systolic MAD measurements

| Measure | Sensitivity | Specificity | Accuracy | PPV | NPV |
| --- | --- | --- | --- | --- | --- |
| Max systolic among 3 views of 5.587 mm | 71%  (10/14) | 69%  (109/159) | 69%  (119/173) | 17%  (10/60) | 96%  (109/113) |
| BSA-indexed max systolic among 3 views of 2.555 mm/m^2^ | 86%  (12/14) | 55%  (87/159) | 57%  (99/173) | 14%  (12/84) | 98%  (87/89) |
| Ht-indexed max systolic among 3 views of 0.033 mm/cm | 79%  (11/14) | 70%  (111/159) | 71%  (122/173) | 19%  (11/59) | 97%  (111/114) |

Ht: height, MAD: mitral annular disjunction.

**Supplemental Table 5:** Univariate model results for the composite outcome

| **Variable** | **Odds Ratio (95% CI)** | **p-value** |
| --- | --- | --- |
| Ht-indexed max systolic MAD among 3 views* | 8.48 (2.26, 31.76) | **0.002** |
| Age at CMR, yr | 0.91 (0.84, 0.98) | **0.013** |
| Male | 0.67 (0.23, 2.01) | 0.479 |
| Medications |  | 0.268 |
| None | 0.47 (0.13, 1.77) |  |
| ≥1 | Ref |  |
| Marfan Syndrome | 0.52 (0.17, 1.57) | 0.244 |
| Early onset, severe Marfan syndrome | 14.18 (2.56, 78.62) | **0.002** |
| Loey-Dietz Syndrome | 3.46 (1.06, 11.28) | **0.040** |
| Ehlers-Danlos syndromes (excluding vEDS) | 3.34 (0.82, 13.63) | 0.093 |
| vEDS | 0.00 (0.00, I) | 0.988 |
| Nonspecific CTD | 0.00 (0.00, I) | 0.966 |
| Other CTD diagnosis | 0.00 (0.00, I) | 0.991 |
| Aortic dissection | 1.67 (0.19, 14.64) | 0.643 |
| Aortic valve surgery | 5.73 (1.81, 18.14) | **0.003** |
| Mitral valve surgery | 2.95 (0.84, 10.34) | 0.091 |
| MV prolapse | 1.82 (0.58, 5.68) | 0.301 |
| TV prolapse | 0.98 (0.29, 3.29) | 0.977 |
| LGE | 0.00 (0.00, I) | 0.988 |
| History of arrhythmia | 2.49 (0.72, 8.64) | 0.150 |
| Max VPB on Holter | 1.13 (1.01, 1.28) | **0.038** |
| Moderate or greater AVVR | 2.31 (0.64, 8.39) | 0.203 |
| LV EDV index, ml/m^2^ | 1.01 (0.99, 1.02) | 0.230 |
| LVEF, % | 0.95 (0.85, 1.05) | 0.306 |
| RV EDV index, ml/m^2^ | 1.01 (0.99, 1.03) | 0.372 |
| RVEF, % | 1.01 (0.93, 1.11) | 0.771 |
| Max ascending aortic Z-score | 1.21 (0.96, 1.53) | 0.114 |
| Max aortic root Z-score | 1.15 (0.91, 1.45) | 0.249 |
| *as a binary predictor with ≥0.033 mm/cm | | |

Patients in which MAD could not be measured in all 3 views were excluded. AVVR: atrioventricular valve regurgitation, CMR: cardiovascular magnetic resonance examination, CTD: connective tissue disorder, EDV: end-diastolic volume, EF: ejection fraction, Ht: height, LGE: late gadolinium enhancement, LV: left ventricle, MAD: mitral annular disjunction, MV: mitral valve, RV: right ventricle, TV: tricuspid valve, VEDS: vascular Ehlers-Danlos syndrome, VPB: ventricular premature beats.

**Supplemental Table 6**: Interobserver agreement

|  | N | Reader 1 (mm) | Reader 2 (mm) | Mean difference ± SD (mm) | ICC (95% CI) |
| --- | --- | --- | --- | --- | --- |
| 2CH diastole | 65 | 1.93 ± 2.50 | 3.22 ± 2.06 | 1.29 ± 1.60 | 0.73 (0.67, 0.78) |
| 4CH diastole | 68 | 1.18 ± 2.04 | 2.43 ± 1.94 | 1.26 ± 1.71 | 0.56 (0.47, 0.64) |
| LVOT diastole | 84 | 1.07 ± 1.92 | 2.33 ± 2.15 | 1.27 ± 1.90 | 0.52 (0.43, 0.61) |
| 2CH systole | 68 | 4.35 ± 4.16 | 4.75 ± 3.16 | 0.40 ± 2.64 | 0.76 (0.71, 0.81) |
| 4CH systole | 69 | 3.58 ± 3.38 | 3.99 ± 2.94 | 0.40 ± 2.15 | 0.81 (0.76, 0.85) |
| LVOT systole | 85 | 3.70 ± 3.91 | 3.61 ± 3.09 | -0.09 ± 1.94 | 0.89 (0.86, 0.91) |

The mean difference is defined as reader 2 minus reader 1. CI: confidence interval, ICC: intraclass correlation coefficient, SD: standard deviation.

**Supplemental Table 7**: Interobserver agreement in the earlier and later era

|  | Earlier era (N=49) | | Later era (N=205) | |
| --- | --- | --- | --- | --- |
|  | N | ICC (95% CI) | N | ICC (95% CI) |
| 2CH diastole | 9 | 0.75 (0.37, 0.92) | 56 | 0.74 (0.62, 0.83) |
| 4CH diastole | 10 | 0.54 (0.02, 0.83) | 58 | 0.64 (0.48, 0.75) |
| LVOT diastole | 10 | 0.15 (-0.42, 0.63) | 74 | 0.68 (0.53, 0.78) |
| 2CH systole | 10 | 0.80 (0.47, 0.94) | 58 | 0.71 (0.57, 0.80) |
| 4CH systole | 9 | 0.86 (0.60, 0.95) | 60 | 0.75 (0.63, 0.83) |
| LVOT systole | 10 | 0.84 (0.56, 0.95) | 75 | 0.86 (0.79, 0.91) |

The earlier era was defined as on or before 12/31/2005 and the later era was defined as 01/01/2006 or later. CI: confidence interval, ICC: intraclass correlation coefficient.

**Supplemental** **Figure 1**: Maximum of systolic MAD measurement (absolute value) by composite outcome status


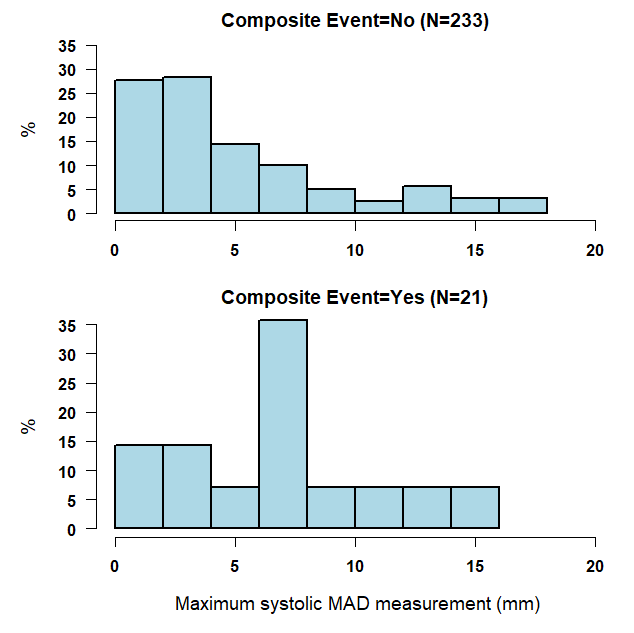

Supplement: Supplementary file 1 — Supplementary material [file mmc1.docx]
