# Supplementary material for: Mitral annular disjunction distance is associated with adverse outcomes in children and young adults with connective tissue disorders
Source: J Cardiovasc Magn Reson. 2025 Sep 6;27(2):101954. doi: 10.1016/j.jocmr.2025.101954 (PMC12702100; doi:10.1016/j.jocmr.2025.101954)
Supplement: Supplementary file 2 — Supplementary material [file mmc2.docx]

**Castellanos et al.**

**SAS CODE**

**SELECTED ANALYSES**

*ANCOVA comparison of CTD cases and controls*

**proc** **glm** data=both;

class dx;

model _2chmvsyst--lvlongaxis = dx htcmr1/ solution;

lsmeans dx/ pdiff stderr;

*Identify predictive threshold for composite clinical endpoint for each MAD measure*

**%macro** hp(x);

proc hpsplit data=clinmad;

class composite;

model composite= &x;

grow entropy;

prune costcomplexity (leaves=**2**);

run;

**%mend**;

*Age-adjusted comparison of Height-indexed MAD for CTD cases with vs. without the composite clinical event*

Let p=Prob(composite event=1)

1-p= Prob(composite event=0)

Logistic Regression Model Statement:

Logit[p/(1-p)] = ß_0_ + ß_1_x_1_ + ß_2_x_2_

Where x_1 =_ Age at CMR in years and x_2_ = MAD measure

**%macro** co(x);

proc logistic data=clinmad descending;

model composite = &x agecmr1;

oddsratio &x;

units &x=**0.005**;

run;

**%mend**;

*Covariate-adjusted model for the composite clinical outcome including the echocardiographic z-scores (key predictor maximum of height-indexed systolic MAD across all 3 views)*

**proc** **logistic** data=clinmad descending;

model composite= binary_maxmadi3ht_sys agecmr1 aosurgery maxvpb tvprol ascaoz arz;
